# Investigations on Fungi Isolated from Apple Trees with Die-Back Symptoms from Basilicata Region (Southern Italy)

**DOI:** 10.3390/plants11101374

**Published:** 2022-05-21

**Authors:** Stefania Mirela Mang, Carmine Marcone, Aurel Maxim, Ippolito Camele

**Affiliations:** 1 School of Agricultural, Forestry, Food and Environmental Sciences (SAFE), University of Basilicata, Viale dell’Ateneo Lucano 10, 85100 Potenza, Italy; 2 Department of Pharmacy, University of Salerno, Via Giovanni Paolo II 132, 84084 Salerno, Italy; cmarcone@unisa.it; 3 Department of Engineering and Environmental Protection, Faculty of Agriculture, University of Agricultural Sciences and Veterinary Medicine, No. 3-5, Calea Manastur Street, 400372 Cluj-Napoca, Romania; aurel.maxim@usamvcluj.ro

**Keywords:** apple die-back, canker, fungi, multi-loci phylogeny, wood decay

## Abstract

Val d’Agri is an important orchard area located in the Basilicata Region (Southern Italy). A phenomenon affecting cv. “Golden Delicious” apples which lead to tree death has been observed in the past several years in this area. This phenomenon has already been detected in about 20 hectares and is rapidly expanding. The symptoms observed were “scaly bark” and extensive cankers, mainly located in the lower part of the trunk, associated with wood decay. Dead plants ranged from 20% to 80% and, in many cases, trees were removed by farmers. In order to identify the causes of this phenomenon, investigations were started in autumn/winter 2019. In order to determine the possible causal agents, fungal and bacterial isolations, from symptomatic tissues, were performed in laboratory. Bacterial isolations gave negative results, whereas pure fungal cultures (PFCs) were obtained after 3–4 passages on potato dextrose agar (PDA) media. Genetic material was extracted from each PFC and amplified by PCR using three pairs of primers: ITS5/4, Bt2a/Bt2b and ACT-512F/ACT-783R. The amplicons were directly sequenced, and nucleotide sequences were compared with those already present in the NCBI GenBank nucleotide database. All isolated fungi were identified based on morphological features and multilocus molecular analyses. *Neofusicoccum parvum*, *Diaporthe eres* and *Trametes versicolor* were most frequently isolated, while *Pestalotiopsis funerea*, *Phomopsis* spp. and *Diaporthe foeniculina* were less frequently isolated. All nucleotide sequences obtained in this study have been deposited into the EMBL database. Pathogenicity tests showed that *N*. *parvum* was the most pathogenic and aggressive fungus, while *Phomopsis* sp. was demonstrated to be the less virulent one. All the investigated fungi were repeatedly reisolated from artificially inoculated twigs of 2-year-old apple trees, cv. “Golden Delicious”, and subsequently morphologically and molecularly identified. The role played by the above-mentioned fungi in the alterations observed in field is also discussed.

## 1. Introduction

Apple die-back syndrome is a complex disease initially characterized by a stunted appearance of the plants and the presence of chlorosis symptoms on leaves. As the disease develops, cracks and necrotic lesions of the cortex appear mostly at the base of the stem and at the grafting point. Subsequently, “scaly bark” and extensive cankers, generally located in the lower part of the trunk develop, which are also associated with wood decay, and the progressive death of the trees is registered. Many authors worldwide investigated the apple die-back syndrome, attributing it to different causal agents, such as various phytopathogens or other possible physiological causes [1,2,3,4,5]. Furthermore, in 2011, Cloete et al. [6] reported the presence of the die-back syndrome and cankers on apples and pears in South Africa, from which they isolated several fungal pathogens belonging to the *Diplodia*, *Phaeoacremonium*, *Phomopsis*, *Neofusicoccum* and *Eutypa* genera. A very recent study by Di Francesco et al. [5], which characterized, in Brazil, apple cultivars’ susceptibility to *Neofusicoccum parvum* (Pennycook & Samuels) P.W. Crous, Slippers & A.J.L. Phillips, stated that due to climate change, this fungus is emerging as a new pathogen on species of the *Rosaceae* plant family. An apple tree die-back syndrome causing severe tree losses was also observed in the main apple producing regions in Tunisia, as described by Souli et al. [7]. The authors identified, both morphologically and molecularly, *Phytophthora* and *Pythium* species as being the causal agents and the factors that promoted apple tree die-back. They also showed that soil salinity contributed to increase the disease severity [7].

In Italy, apple cultivation is mainly concentrated in the northeast part of the country, specifically in Trentino Alto Adige/Südtirol region. This area comprises about half of the harvested hectares and has an intensive growing system, producing almost 70% of the Italian apples. However, some other Italian regions such as Veneto, Emilia-Romagna, Piedmont, Campania and Basilicata also successfully cultivate apple trees with quite relevant productions. Overall, in 2019 in Italy, the surface cultivated with apple was about 58,000 hectares and the apple production reported in the same year was about 23 million quintals [8].

Due to the economic importance of this crop in Italy, many studies regarding its cultivation and phytosanitary status have been performed. The phytosanitary surveys on apples cultivated in Bolzano area, was investigated by Lindner [9]. The author reported the cortical damage of apple cvs. “Gala”, “Golden Delicious” and “Red Delicious”, in spring 2007, and alterations very much resembling the “blister bark” and “paper bark” symptoms often associated the with withering and drying of the branches were also described. Nevertheless, the author reported that they did not isolate any bacterial or fungal pathogen from the plants, concluding that the cause of the observed symptoms was probably to be found among particularly unfavorable climatic conditions registered during the winter and spring periods. The same author also observed damages at the grafting point level and identified two fungi, known as canker agents, namely *Phomopsis mali* and *Diplodia malorum*.

The apple tree die-back syndrome was reported during 2008–2009 in many apple orchards in the north of Italy and since that period many other trees have become infected, especially young plants. 

The phytosanitary status of the apples from Trentino region (Northern Italy) was investigated by Prodorutti et al. [10]. The authors reported an increase in the die-back symptoms on apple trees, showing that plants were usually stunted with cracking and necrosis in the lower part of the trunk and on the graft union site. The trees died during the growing season. Furthermore, the same authors reported that the incidence of the disease was, in some cases, very high (reaching almost 80%), and that the most affected trees were the youngest ones of about 2–5 years old that had been subjected to various types of stress. One bacterium, *Pseudomonas syringae* pv. *syringae*, and a few fungi, such as *Phomopsis* spp., *Neonectria* spp. And some *Botryosphaeriaceae*, were isolated from trunk tissues, taken from symptomatic trees. Their role in the syndrome expression was also demonstrated, even if they appeared not to cause the death of the artificially inoculated branches [10].

The Val d’Agri area, located in the Basilicata Region (Southern Italy), has geographical and climatic conditions that favor apple cultivation. In particular, “Fuji” and “Golden Delicious” apple cultivars are mostly grown in the area. Apple orchards belong to private farmers and are kept as small-scale cultivation systems. During the autumn/winter 2019, in the Val d’Agri area, on about 20 hectares cultivated with apples, symptoms characterized by “scaly bark” and extensive cankers, mainly located in the lower part of the trunk and associated with wood decay, were observed. Additionally, dead plants ranged from 20 to 80%, and in many cases, trees were removed by farmers. In order to identify the causes of this phenomenon, investigations were started in 2019. It is to be mentioned that despite good prevention and control measures employed so far against the key diseases on fruit trees in the south of Italy, including the Basilicata region, knowledge about the distribution and the pathogens involved in apple die-back syndrome is still missing. These data are very important since the presence of the die-back syndrome could economically affect the growers in the region. The identification and characterization of various fungal and bacterial pathogens attacking fruit trees, including apples, were initially based on only the morphological features of the pure cultures obtained in vitro [11,12]. However, over time, despite the ease of application, the morphological features proved to be inefficient to further classify fungal and bacterial pathogens. Therefore, other solutions, including molecular approaches, were investigated in order to identify and characterize the phytopathogens associated with the die-back symptoms [6,7,13,14]. Nowadays, several gene regions or genes, such as the Internal Transcribed Spacer (ITS) of the ribosomal DNA (rDNA), β-tubulin (*TUB-2*) and actin (*ACT*) protein-coding genes, are extensively utilized to identify and characterize phytopathogens [15,16,17,18,19,20,21,22,23]. 

The aim of the present study was to investigate the die-back syndrome on apple orchards from the Val d’Agri area (Basilicata region, Southern Italy). More precisely, the main objectives of the present study were to: (1) identify fungi or bacteria eventually associated with the die-back symptoms observed on apple trees; and (2) perform pathogenicity tests on apple trees in order to verify the involvement of the identified pathogens in the apple die-back disease observed in the Val d’Agri area.

## 2. Results

### 2.1. Pathogens Isolations

Pure culture fungal isolates on PDA media from die-back symptomatic material obtained in this study were selected for further characterization through morphological and cultural characteristics, DNA sequencing and phylogenetic analysis (Table 1).

From apple die-back symptomatic samples, the above-described fungi were isolated with different frequencies. Among the most frequently isolated fungi were the *N. parvum*, with a 55% isolation frequency (IF%), followed by *D*. *eres*, with a 15% IF, and *T*. *versicolor*, with a 14% IF. All the other fungi were less frequently isolated with an IF ranging from 10–12%, except for *Phomopssis* spp., which was very rarely isolated (<5% IF).

Despite repeated trials to isolate bacteria from symptomatic apple wood, no bacterial colonies were ever obtained. During the investigation for the identification of the apple die-back disease cause no symptom or damage of the root system were noticed. Furthermore, all isolation attempts, performed from roots taken from the symptomatic apple trees, gave negative results.

### 2.2. Morphological Identification

**Figure 1:**
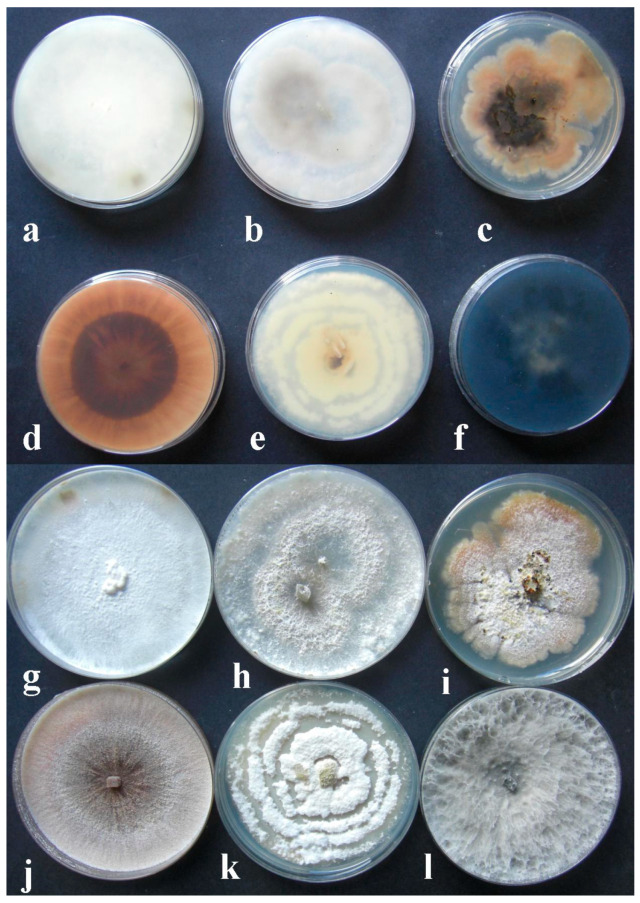
Pure fungal cultures on PDA obtained from samples of apples with dieback symptoms. (**a**,**g**) = *Trametes versicolor*; (**b**,**h**) = *Diaporthe eres*; (**c**,**i**) = *Diaporthe feoniculina*; (**d**,**j**) = *Pestalotiopsis funerea; (***e**,**k**) = *Phomopsis* spp.; (**f**,**l**) = *Neofusicoccum parvum*.

Based on their cultural and morphological features, pure fungal isolates were classified in five distinct genera: *Neofusicoccum* [24], *Diaporthe* [25,26,27,28], *Trametes* [29,30,31,32,33], *Pestalotiopsis* [34,35,36] and *Phomopsis* [6,37,38,39,40] (Table 1 and Figure 1). In particular, in the case of *Diaporthe* grayish or white colonies on PDA and alpha and beta conidia were observed; in the case of *Neofusicoccum*, grey-black colonies and fusiform conidia, nonseptate when young and biseptate ellipsoidal (partially light brown with a darker middle center) when old, were detected. In the case of *Phomopsis,* white colonies and alpha and beta conidia were observed. For *Pestalotiopsis,* reddish colonies and 4-septate conidia, fusiform to ellipsoid and straight to slightly curved, were noticed. *Trametes* genus was identified based on white colonies and the presence of clavate basidia with an inflated epibasidial segment, 4-spored, clamped at the base and basidiospore cylindrical in large spores with slightly inflated top, ellipsoid to ovoid.

### 2.3. Molecular Characterization

The PCR amplifications for each gene investigated yielded amplicons of expected sizes: ITS5/ITS4 (~700 bp), *tub-2* (~500 bp) and *ACT* (~300 bp), which, after direct sequencing in both directions, using the same primers as for the amplification, led to 26 nucleotide sequences (Table 1). A megablast search, excluding “uncultured/environmental sample sequences”, performed in the NCBI’s nucleotide database (www.ncbi.org, accessed on 12 January 2022) for all nucleotide sequences obtained in this study, identified at Genus level all fungal isolates (Table 1).

### 2.4. Phylogenetic Analysis

Single locus analysis gave consistent results for all three loci (ITS, *tub-2* and *ACT*), and the topology of trees was congruent in terms of species grouping. All sequences obtained in this study have been deposited in the European Molecular Biology Laboratory (EMBL-EBI) nucleotide database (www.ebi.ac.uk, accessed on 12 January 2022) and their GenBank accession numbers are presented in Table 1.

**Figure 2:**
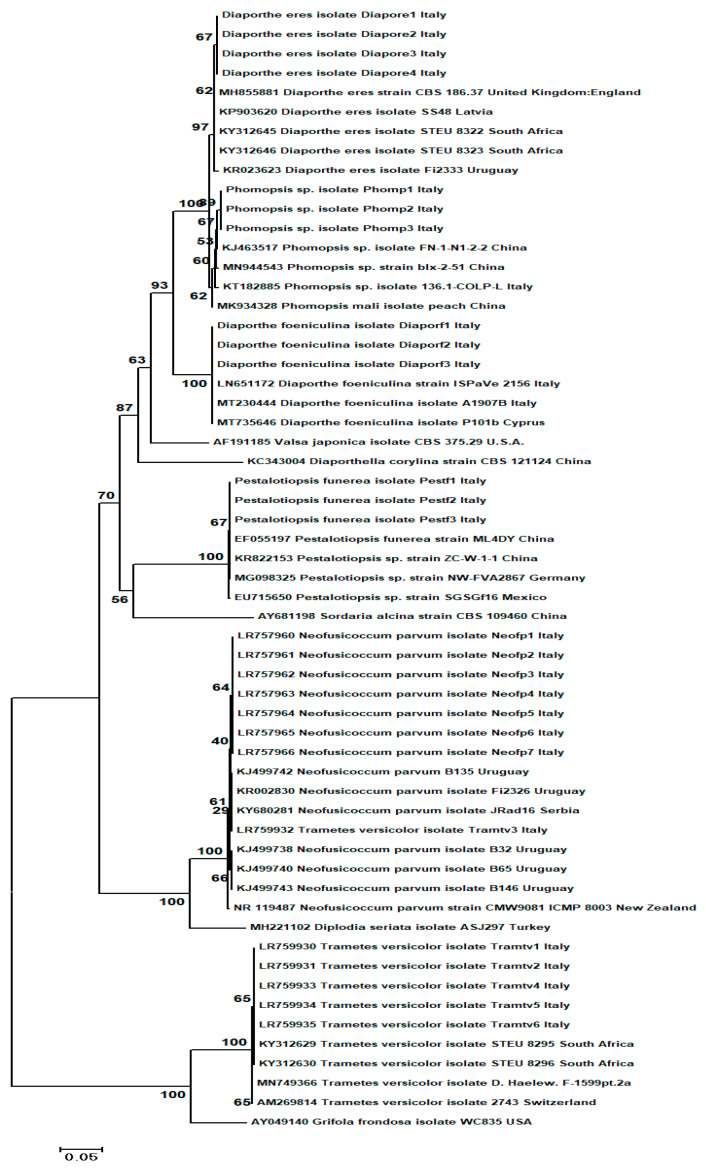
Molecular phylogenetic tree obtained through the neighbor-joining (NJ) method, based on the 58 ITS region sequences data (658 bp) from fungal isolates in the present study and published sequences. Five fungal species (*Diaporthella corylina*, *Valsa japonica*, *Sordaria alcina*, *Grifola fondosa* and *Diplodia seriata*) were used as outgroups in the analysis. The optimal tree with the sum of branch length = 1.38532669 is shown. The confidence probability estimated using the bootstrap test (1000 replicates) is shown next to the branches. The tree is drawn to scale, with branch lengths in the same units as those of the evolutionary distances used to infer the phylogenetic tree. The evolutionary distances were computed using the Tajima–Nei method and are in the units of the number of base substitutions/site. Scientific names of the fungi along with collection place, isolate abbreviation and GenBank AC number are shown in the trees.

The final alignment dataset, for the ITS region, was composed of a total number of 658 characters. It contained 58 nucleotide sequences, including five outgroup species, namely: *Diaporthella corylina* (acc. no. KC343004) utilized for *Diaporthe* fungi; *Valsa japonica* (acc. no AF191185) for *Phomopsis* sp. fungi; *Sordaria alcina* (acc. no. AY681198) for the *Pestaliopsis* sp. Fungal group; *Grifola frondosa* (acc. No. AY049140) utilized for *Trametes* sp. Fungi; and for the *Neofusicoccum parvum* group, *Diplodia seriata* (acc. no. MH221102). Phylogenetic analysis based on the ITS region variation showed that fungal isolates from the same species clustered together into the same clade and, as expected, outgroup species were placed separately from the other groups (Figure 2).

Overall, the 53 nucleotide sequences obtained in this study, based on the ITS region sequence analysis, clustered in two clades which contained all six fungal species. Two of these species belonged to *Diaporthe* genus, namely, *Diaporthe eres* Nitschke and *D. foeniculina* (Sacc.) Udayanga & Castl., and others have been identified as *N. parvum*, *Pestalotiopsis funerea* (Desm.) Steyaert, *Phomopsis* sp. Sacc. & Roum. and *T. versicolor* (L.) Lloyd (Figure 2). Within the phylogenetic tree, the first clade grouped together five of the species previously mentioned, while the second clade contained only one species, *Trametes versicolor*, clearly separated from the others. Additionally, the fungal species isolated and identified in this study were positioned close to similar reference species downloaded from the GenBank for each fungus, and their location was well supported by very high (97–100%) bootstrap values (Figure 2). The ITS data confirmed the previous preliminary fungal identification based on morphological features.

**Figure 3:**
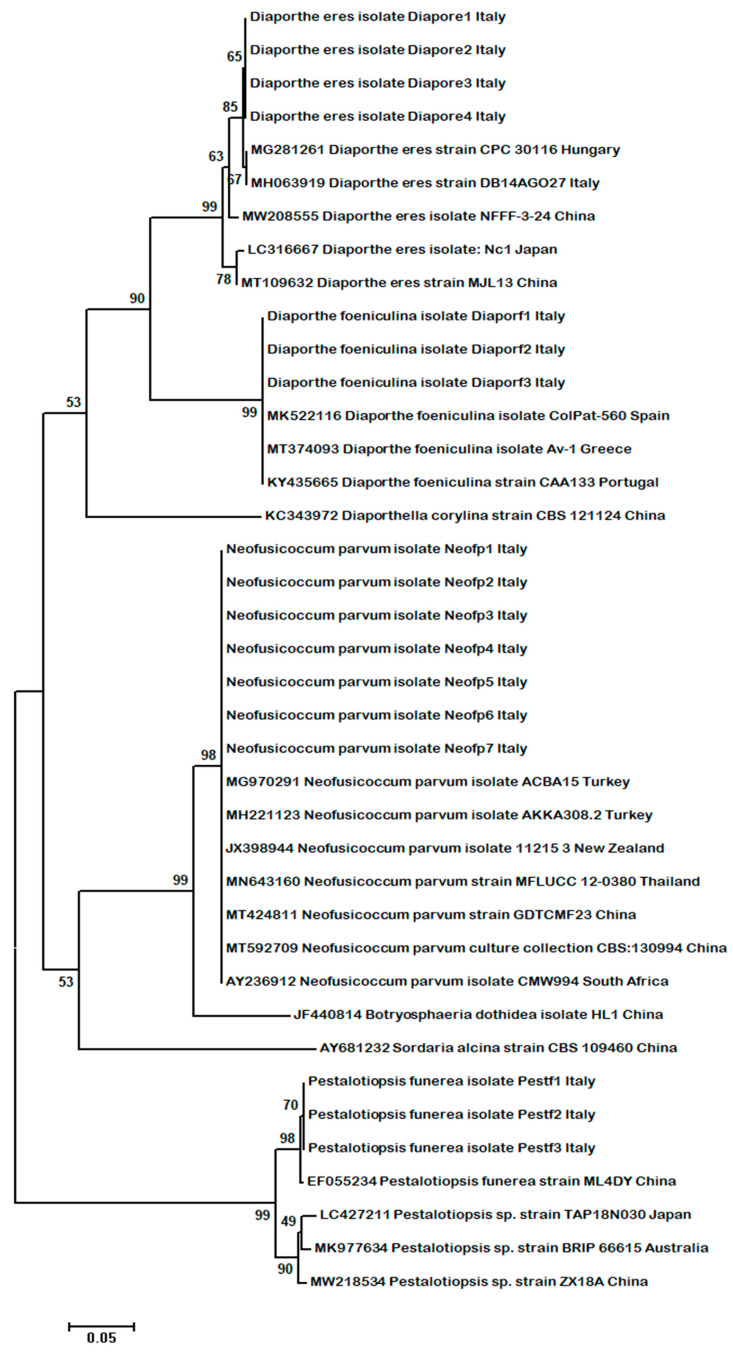
Molecular phylogenetic tree obtained through the neighbor-joining (NJ) method, based on the 39 *tub-2* gene sequences data (500 bp) from fungal isolates in the present study and published sequences. The fungal species (*D. corylina*, *B. dothidea* and *S. alcina*) were used as outgroups in the analysis. The optimal tree with the sum of branch length = 1.08594872 is shown. The confidence probability estimated using the bootstrap test (1000 replicates) is shown next to the branches. The tree is drawn to scale, with branch lengths in the same units as those of the evolutionary distances used to infer the phylogenetic tree. The evolutionary distances were computed using the Tajima–Nei method and are in the units of the number of base substitutions/site. Scientific names of the fungi along with collection place, isolate abbreviation and GenBank AC number are shown in the trees.

Since ITS alone does not provide sufficient resolution to exactly classify fungi at species level, other loci were considered for the phylogeny-based identification of the taxa investigated in this study. In particular, the β-tubulin (*tub-2*) gene, a very well-known molecular locus extensively used in phylogenetic studies of phytopathogenic fungi [35,38,39,40], was examined. A total number of 39 nucleotide sequences of the *tub-2* partial gene were obtained and employed along with the species *D. corylina*, *B. dothidea* and *S. alcina*, used as outgroups in phylogenetic analysis, which was carried out using the NJ method, as performed for the ITS (Figure 3).

Results from the phylogenetic analysis of the fungal species from this study, based on the *tub-2* gene, have shown that they grouped together with similar species from the GenBank database. A better separation within the clades and subclades compared to what obtained from the ITS was also observed (Figure 3). Two separate clades were obtained from the nucleotide sequences investigated in this study. In one, *Neofusicoccum* isolates grouped together into the same subgroup, which was very well supported (98% bootstrap support value), and were separated from the *Pestalotiopsis* subclade, which was also highly supported (99% bootstrap support value), whereas the *Diaporthe* isolates were all placed in a separate clade and were clearly distinguished in two subclades as species, e.g., *D. eres* and *D. foeniculina*, both sharing their vicinity with the same outgroup, *D. corylina* (Figure 3). All fungal species based the *tub-2* gene variation were grouped together with the same species from the GenBank with an elevated bootstrap support (98–99%). The separation of the fungal species within each clade or subclade, strongly supported by high bootstrap values, was 99% for *D. eres*, *D. feoniculina* and *N. parvum* and 98% in the case of *P. funerea* (Figure 3).

**Figure 4:**
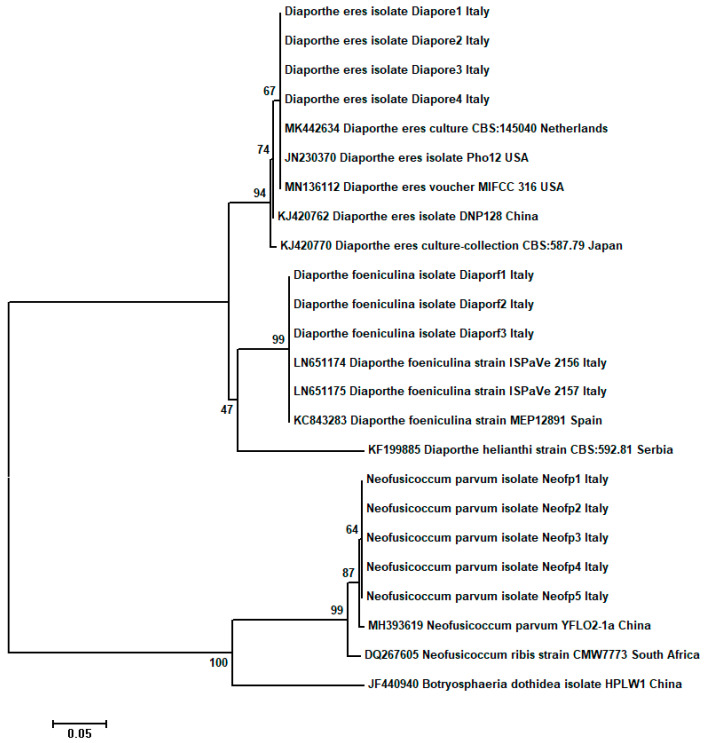
Molecular phylogenetic tree obtained through neighbor-joining (NJ) method based on the 24 *ACT* gene sequences data (298 bp) from fungal isolates in the present study and published sequences. The fungal species (*D. helianthi* and *B. dothidea*) were used as outgroups in the analysis. The optimal tree with the sum of branch length = 0.90526841is shown. The confidence probability estimated using the bootstrap test (1000 replicates) is shown next to the branches. The tree is drawn to scale, with branch lengths in the same units as those of the evolutionary distances used to infer the phylogenetic tree. The evolutionary distances were computed using the Tajima–Nei method and are in the units of the number of base substitutions/site. Scientific names of the fungi along with collection place, isolate abbreviation and GenBank AC number are shown in the trees.

Regarding the third gene, namely actin, despite our repeated PCR trials, amplicons could not be obtained for all fungal species (Figure 4). Therefore, the alignment for the *ACT* gene used in phylogenetic analysis contained 298 characters and involved only 24 nucleotide sequences. Moreover, *ACT* gene analysis showed that the two *Diaporthe* species identified in this study along with their reference species from the GenBank were well separated from the *Neofusicoccum* sp. isolates and, thus, clustered in two different clades also supported by very high bootstrap values of 94% and 99%, respectively. In addition, *Neofusicoccum* isolates were grouped together with their reference species with a 99% bootstrap support value (Figure 4). The phylogenetic reconstruction based on the *ACT* gene reconfirmed the molecular identification based on other loci at species level for each fungal species analyzed and also was in concordance with the preliminary morphological characterization.

Multilocus phylogenetic analyses for *Diaporthe*, *Neofusicoccum* and *Pestalotiopsis* spp. isolates showed that the topology of the trees was congruent in terms of grouping for all fungal species investigated, supporting the single locus phylogenetic outcomes (Figures S1–S3 in Supplementary Material).

### 2.5. Pathogenicity Trial

**Figure 5:**
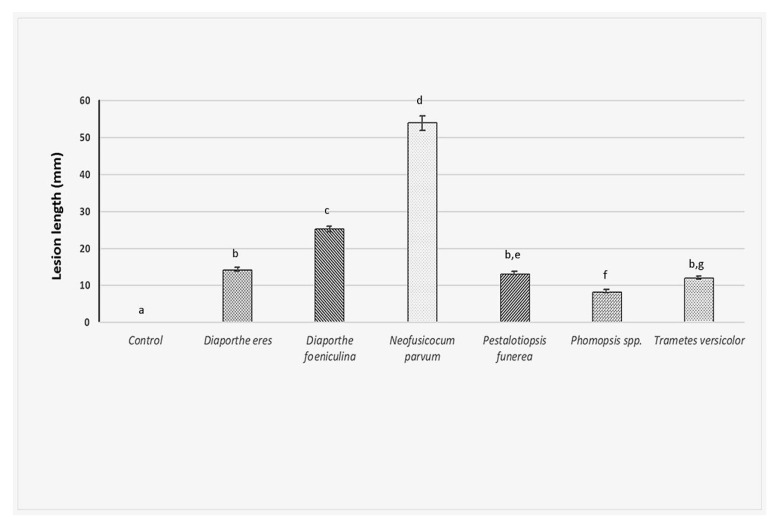
Results of artificial inoculation of the apple twigs with the six fungal isolates investigated. The length of the lesions developed was measured 1 month after inoculation. The experiment was carried out only once with three replications (twigs) and four wounds per replicate. Columns indicate the average length of the lesions with standard errors. Means followed by different letters are significantly different according to Tukey’s test (*p* = 0.01).

In the artificial inoculations test, on twigs of 2-year-old apple trees (cv. “Golden Delicious”), using the six fungi investigated in this study, the size of the observed lesions greatly varied among isolates (*F*_6,77_ = 390, *p* < 0.001). Furthermore, the tested fungal isolates produced lesions in the host that were always larger than those observed from the control (Tukey *t*-tests, *p <* 0.001 in all cases). Most of the fungal isolates (*D*. *eres*, *P*. *funerea*, *Phomopsis* spp. and *T*. *versicolor*) developed smaller lesions than *D*. *foeniculina* and *N*. *parvum*. No lesion developed after control treatment (Figure 5). 

Among all fungi investigated, *N. parvum* produced the longest lesions (53.98 mm). It proved to be also the most pathogenic since the inoculated tree showed very strong die-back symptoms, such as reddish-brown cankers on the twigs, associated with internally brown necrosis. Finally, the death of all twigs and whole branches was observed at one month after artificial infection. *D*. *feoniculina* produced 25 mm length cankers on twigs with internally brown necrosis and death of some twigs and branches was also noticed. *D*. *eres*, *P*. *funerea* and *Phomopsis* formed similar lesions as described above but of a shorter length, which ranged between 8–14 mm. In the case of *T*. *versicolor*, symptoms of wood caries were also seen (Figure 5).

All the inoculated fungi were always reisolated from the lesions and based on molecular methods were identical to the cultures used for inoculation. 

## 3. Discussion

This study is the first to address the presence of the die-back syndrome on apple orchards in the Val d’Agri region and to isolate and further characterize fungal species which could be involved in the observed disease through morphology, DNA sequencing and phylogenetic analysis. The search for the causal agent of the apple die-back syndrome contributed to in vitro isolation of six fungi, already known to be involved in different diseases in apple and other plant species. Both cultural and morphological features of the five phytopathogenic genera, namely *Neofusicoccum*, *Diaporthe*, *Trametes*, *Pestalotiposis* and *Phomopsis*, identified in the present study were consistent and resembled the above-mentioned ones. 

The morphological classification of fungi is an inexpensive and rapid tool but has also many limitations. As a consequence, current mycotaxonomy has changed a lot, now employing other methodological approaches, such as phylogeny, chemotaxonomy, genetics, ecology or molecular biology [35,41,42,43,44,45,46]. The preliminary identification of the fungi isolated from the apple plant, in the present study based on morphological features, was confirmed by molecular outcomes gained from the sequencing of the ITS region, (*tub*-2 and actin (*ACT*) genes. A lesser variation was noticed over the ITS region, for all fungal species investigated, and only in the case of the reference sequences downloaded from the database, which was probably due to sequencing errors. It is widely accepted that sometimes the sequences deposited in the GenBank are of poor quality and around 30% of the ITS sequences deposited may be associated with the wrong taxon [47]. 

ITS locus alone, despite its advantages and official recognition as a DNA barcoding marker [48,49,50,51,52], can be limited in providing enough resolution in the case of closely-related fungal species [53]. Considering all these limitations, for an accurate fungal species identification other loci like *tub*-2 and *ACT* were explored, showing that nucleotide variation was higher in *ACT* gene, followed by the *tub*-2 gene for all fungal species investigated.

Typically, different wood-rotting fungi have been associated with the die-back syndrome in apple over the years as *Coriolus* spp., *Stereum* spp., *Schizophyllum commune* [1]. Apart from these, *Sphaeropsis pyriputrescens* Xiao & J.D Dogers fungus was reported to cause cankers and twig die-back on apple and crabapple trees in the USA [54]. Cloete et al. [6] found that apple and pear trees in South Africa are the hosts of many fungi associated with the die-back symptoms, such as *Diplodia* spp., *Neofusicoccum* spp., *Phaeoacremonium* spp. and *Phomopsis* sp. Very recent studies of Jabiri et al. [55] reported symptoms of dieback disease, such as root rot, yellow leaves and wilting, caused by *Phytopytium vexans* on young apple trees (6–10 years old) of cv. “Golden Delicious” in Morocco. 

The *Diaporthe* genus has also been associated to the shoot canker or fruit rot in pear [56,57]. Dissanayake et al. [28,58], based on molecular phylogenetic analysis, revealed seven new species, within the above-mentioned genus, in Italy. Among the *Diaporthe* species, *D*. *eres* has recently been reported to be linked to necrosis and stem cankers and caused the death of young apple rootstocks in Canada [59]. Moreover, *D*. *eres*, is among the most serious phytopathogenic fungi affecting many plant species all over the world [26,28,56,57,58,59,60,61,62,63]. The outcomes from this study, showing the frequent isolation from apple with die-back symptoms of *D*. *eres,* agree with the previous studies by Sessa et al. [64] who reported the *D*. *eres* isolation from peach and apple with wood disease symptoms, such as wedge-shaped necrosis and canker. Additionally, the identification of more than one species of *Diaporthe*, namely *D*. *eres* and *D*. *neotheicola*, on the same plant species in our study is in agreement with the earlier works of van Niekerk et al. [40] and of Thompson et al. [65], who reported that the same host plant may be colonized by different *Diaporthe* spp. at the same time. 

*Neofusicoccum parvum*, which recently emerged as a phytopathogen, was also among the most frequently isolated fungi in the present study. The *Neofusicoccum* Crous, Slippers and A.J. L. Phillips Genus was described by Crous et al. [24], aiming to include *Botryosphaeriaceae* with *Fusicoccum*-like anamorphs [66]. This fungus has been already reported to cause cankers on many cultivable plant species [67], including apple and pear [68], but it seems that it is expanding its host range, as demonstrated by the very recent study by Choi et al., in Korea [69]. Moreover, *N*. *parvum* was the most aggressive fungus in our study, as demonstrated by both natural and artificial inoculations on apple from Val d’Agri, also causing the death of the entire tree. 

*Trametes versicolor* was among the wood fungi frequently isolated in the present investigation. Fungi from the Genus *Trametes* are white rot polypores. Nearly 60 species are known worldwide on many hosts, and some are used for medicinal purposes [70,71,72], The taxonomy situation within *T*. *versicolor* is still complex, since unresolved phylogenies and unclear species boundaries exist [31,33]. The study of Kile [73], who examined host-pathogen relationships between the apple tree, *T*. *versicolor* and factors affecting host susceptibility, showed that the fungus was a facultative parasite which caused the white rot of the sapwood and the susceptibility of living wood to fungal decay increased with the age of the tree, due to a natural decline in host plant resistance. The frequent isolation of *T*. *versicolor* from apple trees showing die-back symptoms agree with the study of Darbyshire et al. [74], which associated the die-back of apple trees in Australia to the wood-rotting fungus *T*. versicolor and also showed that it is a low-sugar disease. An association between *Coriolus versicolor* (syn. *T*. *versicolor*) and the die-back disease of apples in Washington state, described by Dilley and Covey, supported the present study outcomes [75]. We can assume that frequent in vitro isolation of *T*. *versicolor* from apple trees obtained in our study and the die-back symptoms observed after natural and/or artificial inoculation to this host can be associated with this disease.

Many studies reported that the *Diaporthe* (*Phomopsis*) fungi has been associated with shoot blight and canker, decay, wilting, necrosis of bark and fruit rot in several fruit tree species worldwide [27,37,76,77,78]. The isolation of *Phomopsis* sp. in this study agrees with the earlier studies by Pertot and Vindimian [79], who reported the diffusion of *P*. *mali,* causing the dieback of young apple trees in Trentino (Northern Italy). Cloete et al. [6] also found three *Phomopsis* sp. isolates from pear and apple exhibiting die-back symptoms and considered them as a possible inoculum source for grapevine trunk disease pathogens. Our outcomes concord with the results of Bai et al. [56], who identified the presence of these fungi on pear in China, and of Kanematsu et al. [37], who, in Japan, showed that they were responsible for shoot cankers. 

Regarding *Diaporthe* species, our results from pathogenicity tests are similar to those reported by Sessa et al. [64], who investigated the diversity and the virulence of the *Diaporthe* species associated with wood disease symptoms in deciduous fruit trees in Uruguay. The same authors recognized them to be the causal agents of twig and branch cankers, showing that *D*. *eres* and *D*. *foeniculina* produced necrosis. Furthermore, another study by Abramczyk et al. [62] characterized isolates of *D. eres* based on morphological and pathological characteristics, which were isolated from fruit plants and genetically identified as *D. eres* species complex [78]. Additionally, they demonstrated that in pathogenicity tests *D*. *eres* produced small necrosis of about 12–17 mm in diameter, occurring at the site of inoculation. *D*. *eres* colonies were obtained from the artificially inoculated tissue, again confirming the results obtained in our study on this fungus, showing its pathogenic abilities towards apple trees.

Pathogenicity tests results showing that *N*. *parvum* was the most virulent among all fungal species isolated and identified on apple trees from the Val d’Agri region match those by Cloete et al. [6]. The authors analyzed fungi associated with die-back symptoms of apple and pear trees cultivated in proximity of grapevine in Western Cape, South Africa, and found that a species of *Neofusicoccum* (*N. australe*) was among the most virulent species towards apple, with mean necrotic lesions of about 40.2 mm length. In the same study, *Phomospsis* sp. was observed to be less virulent (necrotic lesions of about 11.8 mm in length), and this was similar to the results obtained in the present study (necrotic lesions of about 8 mm in length). Another study by Espinoza et al. [80] found that *Neofusicoccum* spp. was associated with the stem canker and dieback of blueberry in Chile and reported, for the first time, *N*. *parvum* as a canker-causing agent on blueberry. In their study, the same authors performed pathogenicity tests on kiwi, blueberry and apple and found that *N*. *parvum* was the most aggressive fungus, in all hosts, and this is also in accordance with our results.

## 4. Materials and Methods

### 4.1. Biologic Material

**Figure 6:**
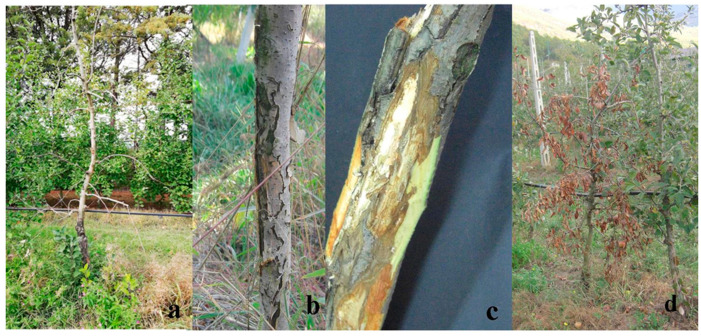
Apple trees located in the Val d’Agri area showing die-back symptoms (**a**–**c**) and death of tree caused by die-back (**d**). Figure a—courtesy of Dr. Camilla Nigro, ALSIA, Basilicata Region.

Pieces of symptomatic trunks from the apple cv. “Golden Delicious”, showing die-back symptoms, were collected in autumn/winter 2019. During this period, apple orchards located in the Val d’Agri area were surveyed for the presence of apple die-back symptoms. A total number of 50 samples, made of pieces of living material (bark and cankered trunks) showing die-back symptoms, more specifically, “scaly bark” and extensive cankers, mainly located in the lower part of the trunk, and wood decay were obtained from trees 3–12 years old (Figure 6). They were brought to the Plant Pathology Laboratory at the University of Basilicata and stored in fridges, at 4 °C, until used. 

### 4.2. Pathogen Isolation

Symptomatic wood pieces were cut under laminar flow sterile conditions into small parts, surface-sterilized by soaking in a 70% ethanol solution for 1 min, in a 1% NaOCl solution for 1 min, in 70% ethanol solution for another 30 sec and finally rinsed in sterile water for 2 min. After sterilization, the trunk pieces were dried on a sterile paper and cut into small parts. Small parts of about 2 × 2 mm taken from the margins between necrotic and healthy tissue were placed on petri plates containing potato dextrose agar (PDA, Oxoid Ltd., Hants, UK), amended with streptomycin sulphate (40 mg L^−1^, MerckKGaA, Darmstadt, Germany) and were incubated at 25 °C in the dark until growth could be detected. Subcultures were performed from the growing hyphae onto PDA and incubated under the same conditions. Pure cultures were created for all obtained PDA plates.

To isolate and identify bacterial pathogens probably linked to the die-back syndrome symptomatic wood trunk, samples were first surface sterilized and prepared, as reported by Schaad et al. [81]. 

### 4.3. Morphological Identification

All fungal isolates obtained in this study were stored, as pure cultures (PFC), in the culture collection of the Plant Pathology Laboratory of the School of Agriculture, Forestry, Food and Environmental Sciences (SAFE) at the University of Basilicata on PDA slants and maintained at 4 °C in fridge.

Fungal isolates were examined using a Axioscope microscope (Zeiss, Jena, Germany) and preliminary identified by morphological characteristics. 

### 4.4. Molecular Characterization

For molecular characterization, genomic DNA was extracted from fresh PFC mycelia of each isolate, 7–10 days old, through an extraction protocol described by Mang et al. [43]. Genomic DNA quality and quantity were checked using a Nanodrop ND-1000 spectrophotometer (Thermo Scientific Inc., Willmington, DE, USA) and the material was stored at −20 °C in 1.5 mL Eppendorf tubes until further use. In order to determine the fungal species, three different genes/regions were amplified. Namely, the internal transcribed spacers (ITS1 and ITS2) of the ribosomal RNA (ITS); β-tubulin (*tub-*2) and actin (*ACT*). The oligonucleotides used for PCR amplifications were: ITS5/ITS4 [82], Bt2a/Bt2b [83] and ACT512F/ACT783R [84] (Table 2).

PCR amplifications were performed under the conditions explained in Mang et al. [43,85] for ITS only. For the other two genes the Phire Direct PCR Master mix (Thermo Scientific Inc., USA) was used, following manufacturer’s instructions with some modifications. PCR mixtures were composed of 10 µL of 2X Phire Plant PCR Buffer (including 1.5 mM MgCl_2_ and 20 µM of dNTPs), Primers 0.5 µM each; 0.4 µL of Phire Hot Start II DNA polymerase enzyme, 5 µL of template DNA (20 ng/µL) and double distilled water up to 20 µL. The PCR cycling protocol consisted of: an initial denaturation at 98 °C for 5 min for 1 cycle; then 40 cycles of denaturation at 98 °C for 5 s; annealing at 60 °C for ITS and at 62 °C for *tub-2* and *ACT* genes for 5 s; extension at 72 °C for 20 s, followed by a final extension at 72 °C for 1 min for 1 cycle. All PCR products were separated in 1.5% agarose gels in Tris-Acetic acid-EDTA (TAE) buffer and visualized under the UV after staining with SYBR Safe DNA Gel Stain (ThermoFisher Scientific™, Carlsbad, CA, USA). A 100-bp GeneRuler Express DNA Ladder (ThermoFisher Scientific™ Baltics UAB, Vilnius, Lithuania) was used as a molecular weight marker. Direct sequencing of all PCR products was performed by BMR Genomics [Padua, Italy], using a 3130xl automatic sequencer in both directions and using the same primers as for the PCR. Subsequently, the sequence information was analyzed by the local alignment search tool using BLASTn [86,87] in the National Center for Biotechnology Information (NCBI) database (http://www.ncbi.nlm.nih.gov/BLAST, accessed on 12 January 2022). Annotations were based on BLAST searches with a minimum of 99–100% identity over at least 80% of the length of the nucleotide sequence, which are the commonly used thresholds for reliable sequence annotation [88]. Nucleotide sequences primary identification was carried out through the BLASTn search tool program [86,87] of the NCBI by comparing all sequences obtained in this study with those already present in the database.

### 4.5. Sequences Alignments and Phylogenetic Analysis

All nucleotide sequences produced by this study and identified based on high sequence identity (>99–100%) to similar species already present in nucleotide databases, along with few additional reference sequences downloaded from GenBank (http://www.ncbi.nlm.nih.gov/GenBank, accessed on 12 January, 2022), were used for the phylogenetic analysis. Subsequently, they were manually edited and aligned with the ClustalX version 2.0 [88] program, using the MEGA X (Molecular Evolutionary Genetic Analysis) [89] phylogeny package to build representative alignments (Table 3). As reported in previous studies by Slippers et al. [66] and by Crous et al. [24], *N. parvum* and *N*. *ribis* are closely related cryptic species within the recently described Genus *Neofusicoccum* (*Botryosphaeriaceae*, *Ascomycetes*). Therefore, in case of the *ACT* gene, when no other reference species were available in the GenBank nucleotide database, this fungal species was also used, allowing us to perform the phylogenetic investigation (Table 3).

Phylogeny reconstructions were performed with MEGAX [89] for each gene using the neighbor-joining (NJ) statistical method [90] with an interior branch test and 1000 bootstrap replications [91,92], nucleotide substitution type and the Tajima–Nei substitution model [93] with uniform rates among sites. A deletion was used as treatment for gaps and missing data and the codon positions included were 1st, 2nd, 3rd and noncoding sites. The evolutionary distances computed using the Tajima–Nei method [93] are in the units of the number of base substitutions/site. The same procedure described previously was used for the *tub-2* and *ACT* genes. An unequal number of nucleotide sequences were involved in the phylogenetic analyses for each gene investigated, which was caused by the lack of positive PCR and sequencing results for some of the genes and also the nonexistence of nucleotide sequences in the GenBank database. Therefore, only single gene phylogenies could be performed, each one containing all nucleotide sequences obtained in this study for the examined gene plus reference species downloaded from the GenBank.

In particular, the reference sequences representing the relevant species used to build alignments for species identification were: for *D. eres* and *D*. *foeniculina* (*D. corylina* strain CBS121124 and only for *ACT* gene *D. helianthi* strain AR4131), *N. parvum* (*Diplodia seriata* strain ASJ297; or *Botryosphaeria dothidea* strains HL1 and HPLW1 for the *tub-2* and *ACT* genes, respectively), *Pestalotiopsis* sp. (*Sordaria alcina* strain CBS 109460), *Phomopsis* sp. (*Valsa japonica* isolate CBS375.29) and *Trametes versicolor* (*Grifola frondosa* isolate WC835) (Table 3). 

A different number of nucleotide sequences were obtained for each gene and fungal species in this study; therefore, a multilocus phylogeny with three genes (*ITS* + *tub-2* + *ACT*) was possible only for *Diaporthe* and *Neofusicoccum* spp., while for *Pestalotiopsis* spp. a two-gene phylogeny (*ITS* + *tub-2*) was performed using the Seaview5 program, as presented in Table S1 and in Figures S1–S3. (Supplementary material).

### 4.6. Pathogenicity Trials

A trial was conducted under field conditions to examine the formation of lesions on twigs of 2-year-old apple trees (cv. “Golden Delicious”), using a common protocol. In particular, the pathogenicity tests were performed using 4 mm diameter mycelial plugs taken from the margins of 7-day-old cultures on PDA amended with antibiotic streptomycin sulphate (40 mg L^–1^, MerckKGaA, Darmstadt, Germany). An equal number of young apple shoots were equally treated but using only sterile agar plugs, which were left as controls. One fungal isolate was used for each apple tree, according to the fungal species identified and characterized in this study, and each treatment was replicated four times. A wound of the about the size of the agar plug was made on each woody shoot, in the phloem and cortex tissue, with a sterile scalpel. Immediately after wounding, the plug was positioned in the center of the wound and covered by a sterile water wetted cotton piece. In order to avoid a rapid dehydration, lesion sites were wrapped with parafilm (Pechiney Plastic Packaging, Menasha, WI, USA). For each fungal pathogen the trial layout was a randomized block design with four repetitions using twigs as experimental units. The whole pathogenicity trial consisted of six fungal pathogens, isolated in this study from apple trees in Val d’Agri, and an agar plug only. Following inoculation, all young apple trees were placed in a greenhouse, where they were kept under natural light conditions at 22 °C and at about 70% relative humidity. After 30 days of inoculation, apple twigs were inspected for lesion development and after 45 days post-inoculation, when their necrosis was evident, the twigs were removed and brought to the laboratory for immediate analysis. The number of twigs with necrosis was recorded and, after the removal of the bark, the length of the developed canker lesions was measured. In order to reisolate the causal agent, small pieces (approx. 5 mm length) of diseased wooden tissue were cut from the edge of the necrotic lesions from the inoculated twigs and, after surface disinfection, were placed in petri dishes containing PDA and antibiotic streptomycin sulphate. Plates were incubated for 7 days at 20 °C in an incubator, under dark conditions, until growth was detected. Subsequently, the identification of the reisolated fungi was carried out by both morphological features and molecular analysis, using the protocols for morphological identification, DNA extraction and PCR conditions described above.

### 4.7. Statistical Analysis

Since the data obtained from the lesion measurements were normally distributed (Shapiro–Wilk tests [94], followed by a Holm–Bonfferoni [95] correction), a one-way ANOVA was used to test for mean differences among the investigated fungal isolates. Tukey *post hoc* tests for multiple comparisons of means were also performed to detect significant differences among the treatments. The statistical analyses performed in this study were performed using the R version 3.6.2 software (R Core Team, Vienna, Austria) [96].

## 5. Conclusions

Fungal species investigated in this study are well known to be involved in the fruit tree trunk diseases. The present study demonstrated that among all fungi investigated, *N*. *parvum* was the most aggressive and may be involved in the heavy decline of apple trees in the Val D’Agri area. In addition, other fungi, such as *D*. *eres*, *D*. *foeniculina*, *P*. *funerea*, *T*. *versicolor* and *Phomopsis* spp., could have contributed to the aggravation of the existing symptoms. Our field observations allowed us to assume that fungi, and in particular *N*. *parvum*, could penetrate the trees through wounds created by cuttings. Therefore, to avoid this, it is necessary to protect the wounds, in particular after cuttings.

Given the economic importance of apples worldwide, more investigations related to the role played by the phytopathogens discovered in this study, which are involved in die-back disease on apple trees, seem necessary. Future outcomes will be expected to add beneficial knowledge to better understand this complex disease in order to establish appropriate strategies to protect this regionally relevant and worldwide nutritionally important crop.

## Figures and Tables

**Table 1:** Fungal isolates obtained during this study with their respective GenBank accession numbers and percentage of identity when compared to reference nucleotide sequences for the same species from the NCBI nucleotide database.

Isolate Name	Species	GenBank Accession Number *			Identity (%) *		
		ITS **	TUB-2 **	ACT **	ITS **	TUB-2 **	ACT **
**Noefp1**	*Neofusicoccum parvum*	LR757960	OU022063	OU023206	>99–100	>99	100
Neofp2	*N*. *parvum*	LR757961	OU022064	OU023207	-//-	-//-	-//-
Neofp3	*N*. *parvum*	LR757962	OU022065	OU023208	-//-	-//-	-//-
Neofp4	*N*. *parvum*	LR757963	OU022066	OU023209	-//-	-//-	-//-
Neofp5	*N*. *parvum*	LR757964	OU022067	OU023210	-//-	-//-	-//-
Neofp6	*N*. *parvum*	LR757965	OU022068	OU023211	-//-	-//-	-//-
Neofp7	*N*. *parvum*	LR757966	OU022069	OU023212	-//-	-//-	-//-
Tramtv1	*Trametes versicolor*	LR759930	-	-	>99–100	-	-
Tramtv2	*T*. *versicolor*	LR759931	-	-	-//-	-	-
Tramtv3	*T*. *versicolor*	LR759932	-	-	-//-	-	-
Tramtv4	*T*. *versicolor*	LR759933	-	-	-//-	-	-
Tramtv5	*T*. *versicolor*	LR759934	-	-	-//-	-	-
Tramtv6	*T*. *versicolor*	LR759935	-	-	-//-	-	-
Diapore1	*Diaporthe eres*	OU020696	OU022056	OU023199	>99	>99–100	>99
Diapore2	*D*. *eres*	OU020697	OU022057	OU023200	-//-	-//-	-//-
Diapore3	*D*. *eres*	OU020698	OU022058	OU023201	-//-	-//-	-//-
Diapore4	*D*. *eres*	OU020699	OU022059	OU023202	-//-	-//-	-//-
Diaporf1	*Diaporthe foeniculina*	OU020700	OU022060	OU023203	>99–100	100	>99
Diaporf2	*D*. *foeniculina*	OU020701	OU022061	OU023204	-//-	-//-	-//-
Diaporf3	*D*. *foeniculina*	OU020702	OU022062	OU023205	-//-	-//-	-//-
Pestf1	*Pestalotiopsis funerea*	OU020703	OU022070	-	>99	>99	-
Pestf2	*P*. *funerea*	OU020704	OU022071	-	-//-	-//-	-
Pestf3	*P*. *funerea*	OU020705	OU022072	-	-//-	-//-	-
Phomp1	*Phomopsis* sp.	OU026160	-	-	>99	-	-
Phomp2	*Phomopsis* sp.	OU026161	-	-	-//-	-	-
Phomp3	*Phomopsis* sp.	OU026162	-	-	-//-	-	-

Note: * The percentage of identity was established after comparing the nucleotide sequences from this study with at least two of the reference species existent in the database for each fungal species. ** ITS = Nuclear ribosomal internal transcribed spacer regions; TUB-2 = β-tubulin 2 gene; ACT = actin gene. “-” = no data were obtained/or exist in the GenBank nucleotide database. “-//-” = identical values as those in the previous row are reported.

**Table 2:** Details of primers pairs used in this study for the amplification and sequencing of fungal DNA.

Locus *	Primer	Sequences 5′→3′	Reference
ITS	ITS5	5′-GGA AGT AAA AGT CGT AAC AAG G-3′	White et al., 1990
	ITS4	5′-TCC TCC GCT TAT TGA TAT GC-3′	
TUB-2	Bt2a	5′-GGT AAC CAA ATC GGT GCT GCT TTC-3′	Glass and Donaldson, 1995
	Bt2b	5′-ACC CTC AGT GTA GTG ACC CTT GGC-3′	
ACT	ACT-512F	5′-ATG TGC AAG GCC GGT TTC GC-3′	Carbone and Kohn, 1999
	ACT-783R	5′-TAC GAG TCC TTC TGG CCC AT-3′	

* ITS: internal transcribed spacer regions and intervening 5.8S rRNA gene; TUB-2: partial beta tubulin gene; ACT: actin gene.

**Table 3:** List of taxa, fungal isolates and GenBank accession numbers of the genes analyzed in this study and used for phylogenetic analysis.

Taxa	Isolate Name	Culture No.	Gene	GenBank Accession Number *			Reference
	ITS	TUB-2	ACT	ITS	TUB-2	ACT	
*Diaporthe eres*	CBS 186.37	CPC 30116	CBS:145040	MH855881	MG281261	MK442634	Wu et al., 2019
*D. eres*	SS48	NEFF 3-23-4	Pho12	KP903620	MW208555	JN230370	GenBank
*D. eres*	STEU 8322	DB14AGO27	MIFCC 316	KY312645	MH063919	MN136112	GenBank
*D. eres*	STEU 8323	Nc1	CBS:587.79	KY312646	LC316667	KJ420770	GenBank
*D. eres*	Fi2333	MJL13	DNP128	KR023623	MT109632	KJ420762	GenBank
*Diaporthe foeniculina*	ISPaVe 2156	ColPat-560	ISPaVe 2156	LN651172	MK522116	LN651174	GenBank ^a^ Lopez-Moral et al., 2020
*D*. *foeniculina*	A1907B	Av-1	ISPaVe 2157	MT230444	MT374093	LN651175	GenBank ^c^ Udayanga et al., 2014
*D*. *foeniculina*	P101b	CAA133	MEP12891	MT735646	KY435665	KC843283	GenBank ^a^ Mathioudakis et al., 2020 ^c^ Udayanga et al., 2014
** *Diaporthella corylina*	CBS121124	CBS121124	CBS:592.81	KC343004	KC343972	N/A	Gomes et al., 2013
** *Diaporthe helianthi*	N/A	N/A	AR4131	N/A	N/A	KF199885	^c^ GenBank
*Neofusicoccum parvum*	B32	ACBA15	YELO-21a	KJ499738	MG970291	MH393619	GenBank
*N. parvum*	B65	AKKA308.2	CMW 7773	KJ499740	MH221123	- ^e^	GenBank ^c^ Hunter et al.,2006
*^d^ N. ribis*	- ^e^	- ^e^	CMW 7773	- ^e^	- ^e^	DQ267605	GenBank
*N. parvum*	B135	11215_3	N/A	KJ499742	JX398944	N/A	GenBank
*N. parvum*	B146	MFLUCC_12-0380	N/A	KJ499743	MN643160	N/A	GenBank
*N. parvum*	Fi2326	GDTCMF23	N/A	KR002830	MT424811	N/A	GenBank
*N. parvum*	JRad16	CBS:130994	N/A	KY680281	^a^ MT529709	N/A	GenBank ^a^ Zhang et al., 2021
*N. parvum*	CMW9081; ICMP 8003	CMW994	N/A	NR119487	^a^ AY236912	N/A	GenBank ^a^ Slippers et al., 2004
** *Diplodia seriata*	ASJ297	HL1	N/A	MH221102	JF4040814	N/A	GenBank
*^b^ Botryosphaeria dothidea*	N/A	N/A	HPLW1	N/A	N/A	JF440940	^a^ Tang et al., 2012
*Pestalotiopsis funerea*	ML4DY	ML4DY	N/A	EF055197	EF055234	N/A	GenBank
*Pestalotiopsis* sp.	SGSGf16	TAP18N030	N/A	EU715650	LC427211	N/A	GenBank
*Pestalotiopsis* sp.	ZC-W-1-1	BRIP 66615	N/A	KR822153	MK977634	N/A	GenBank
*Pestalotiopsis* sp.	NW-FWA2867	ZX18A	N/A	MG098325	MW218534	N/A	GenBank
** *Sordaria alcina*	CBS 109460	CBS 109460	N/A	AY681198	^a^ AY681232	N/A	Liu et al., 2010 ^a^ Cai et al., 2006
*Phomopsis* sp.	FN-1-N1-2-2	N/A	N/A	KJ465317	N/A	N/A	GenBank
*Phomopsis* sp.	blx-2-51	N/A	N/A	MN944543	N/A	N/A	GenBank
*Phomopsis* sp.	Peach	N/A	N/A	MK934328	N/A	N/A	GenBank
*Phomopsis* sp.	136.1-COLP-L	N/A	N/A	KT182885	N/A	N/A	GenBank
^**^ *Valsa japonica*	CBS375.29	N/A	N/A	AF191185	N/A	N/A	Adams et al., 2002
*Trametes versicolor*	D. HaelewF-1599pt.2a	N/A	N/A	MN749366	N/A	N/A	GenBank
*T. versicolor*	STEU 8295	N/A	N/A	KY312629	N/A	N/A	GenBank
*T. versicolor*	STEU 8296	N/A	N/A	KY312630	N/A	N/A	GenBank
*T. versicolor*	2473	N/A	N/A	AM269814	N/A	N/A	GenBank
** *Grifola frondosa*	WC835	N/A	N/A	AY049140	N/A	N/A	Shen et al., 2002

Notes: * ITS-Nuclear ribosomal internal transcribed spacer regions; TUB- (*tub*-2) gene; *ACT*-actin gene. ** This fungal species has been used as outgroup for the phylogenetic analysis. The sign “-” in the column table indicates either that the nucleotide sequences do not exist in the GenBank or were not obtained in this study for that particular fungal isolate. ^a^ This study was considered only for the *tub*-2 nucleotide gene sequence. ^b^ This fungal species was used only for the phylogenetic analysis involving the *tub*-2 gene nucleotide sequences. ^c^ This GenBank origin of the accession regards only *ACT* gene nucleotide sequences used for the phylogenetic analysis. ^d^ This fungal species was used only for the phylogenetic analysis involving the *ACT* gene. N/A: not available.^e^ Data not considered.

## Data Availability

The authors declare that the data supporting the findings of this study are available within the article and in the supplementary materials (Figures S1–S3 and Table S1).

## Supplementary Materials

The following supporting information can be downloaded at: https://www.mdpi.com/article/10.3390/plants11101374/s1, Figure S1: PhyML tree (ITS + *tub-2* + *ACT)* for *Diaporthe* spp.; Figure S2: PhyML tree (ITS + *tub-2* + *ACT*) for *Neofusicoccum* spp.; Figure S3: PhyML tree (ITS + *tub-2*) for *Pestalotiopsis* spp.; Table S1: Multilocus phylogeny parameters used in the study.
