# Supplementary material for: Investigations on Fungi Isolated from Apple Trees with Die-Back Symptoms from Basilicata Region (Southern Italy)
Source: Plants (Basel). 2022 May 21;11(10):1374. doi: 10.3390/plants11101374 (PMC9147037; doi:10.3390/plants11101374)
Supplement: Supplementary file 1 [file plants-11-01374-s001.zip › plants-1642785-supplementary.pdf]

Table S1. Multilocus phylogeny parameters used in the study.

**Parameter**	**Details**
Analysis*	PhyML
Model	GTR
Branch Support	Bootstrap with 1000 replicates
Nucleotide equilibrium frequencies	Empirical
Invariable sites	None
Across site rate variation	Optimized, classes 4
Tree searching operations	NNI
Starting tree	BioNJ, Optimize tree topology

* The analysis was performed with Seaview5 program only for *Diaporthe*, *Neofusicoccum* spp. (*ITS+tub-2+ACT*) and *Pestalotiopsis* spp. (*ITS+tub-2*) isolates obtained in the study along with reference species downloaded from NCBI nucleotide database.

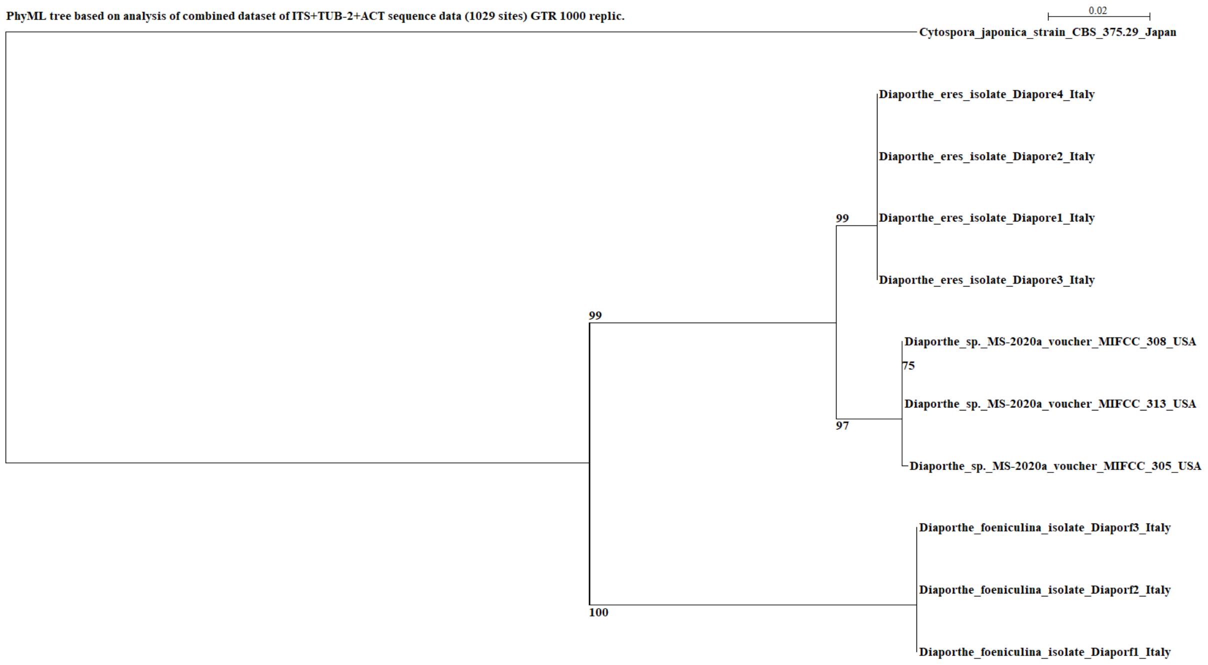

Figure S1.PhyML tree (ITS + tub-2 + ACT) for Diaporthe spp.

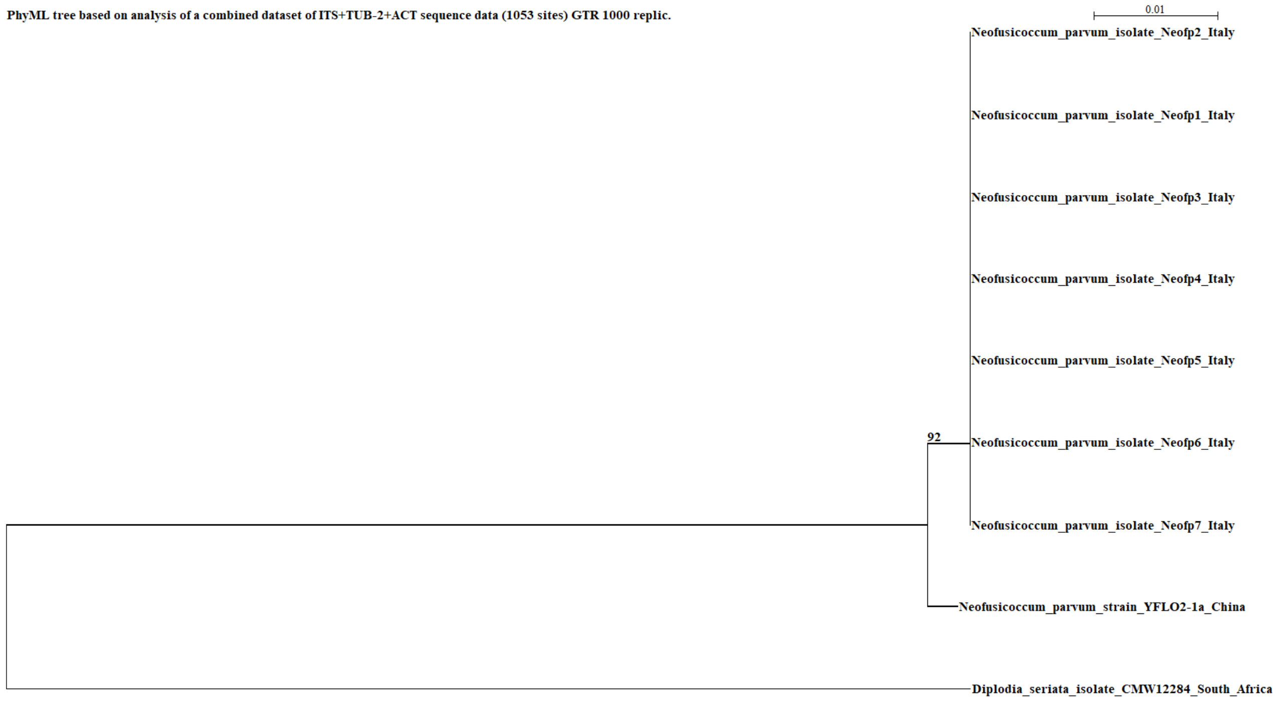

Figure S2.PhyML tree (ITS + tub-2 + ACT) for Neofusicoccum spp.

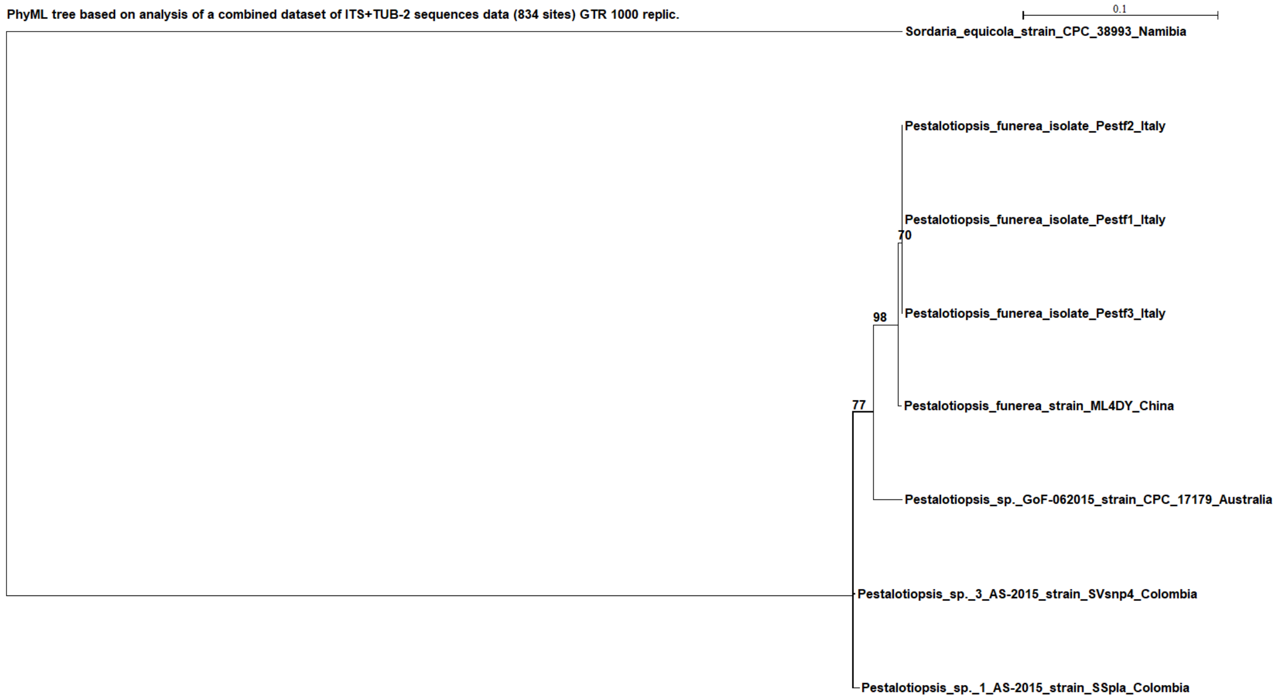

Figure S3.PhyML tree (ITS + tub-2) for Pestalotiopsis spp.
